# Comparing the clinical outcomes of laparoscopic sleeve gastrectomy and hiatal hernia repair with or without fundoplication for weight loss and gastrointestinal reflux resolution

**DOI:** 10.3389/fsurg.2025.1513695

**Published:** 2025-04-03

**Authors:** Hussam Al Trabulsi, Dunia Al Trabulsi, Khadeja Alrefaie, Tala Muassess, Salman Yousuf Guraya

**Affiliations:** ^1^ Department of Surgery, Medcare Hospital Al Safa, Dubai, United Arab Emirates; ^2^ Department of Health Sciences, School of Health and Life Sciences, University of Nicosia, Nicosia, Cyprus; ^3^ School of Medicine, Royal College of Surgeons in Ireland (Bahrain), Al Muharraq, Bahrain; ^4^ Clinical Sciences Department, College of Medicine University of Sharjah, Sharjah, United Arab Emirates

**Keywords:** GERD, obesity, laparoscopic sleeve gastrectomy, hiatal hernia repair, Nissen fundoplication

## Abstract

**Introduction:**

Evidence suggests that hiatal hernia should be repaired if found during laparoscopic sleeve gastrectomy (LSG), either to prevent new-onset post-operative gastro-esophageal reflux disease (GERD), or to treat pre-existing reflux symptoms. There is interest in performing laparoscopic Nissen's fundoplication (LNF) along with hiatal hernia repair (HHR) during LSG. This study aimed to determine whether hiatal crural repair alone is adequate for symptomatic control. We compared operative time, body mass index (BMI), and reflux symptoms between those undergoing LSG with HHR vs. LSG with HHR and LNF.

**Materials and methods:**

We retrospectively analyzed clinical data of patients who underwent LSG with HHR. This cohort was divided into those with LNF (group 1) and without LNF (group 2). We collected patients' pre-operative BMI and GERD Questionnaire (GERD-Q) scores. We then compared pre-operative BMI and GERD-Q values with post-operative indices at 1-month, 3-months, and 6-months. The patients' medical records for operative findings and time between both groups was analyzed. Statistical analyses included Independent Samples *T*-tests, Paired *T*-tests, and correlation analysis.

**Results:**

In this study, 978 bariatric surgeries were performed. Of 431 LSG patients, 73 fulfilled the study criteria. Both groups showed significant reduction in BMI and GERD-Q scores post-operatively. Group 1 had a decrease in BMI from an average pre-operative value of 38.03–32.17 at 6 months (*p* < 0.001), and GERD-Q scores from 12.25 to 6.47 (*p* < 0.001). Group 2 showed a BMI decrease from 39.63 to 31.67 (*p* < 0.001) and GERD-Q scores from 11.54 to 6.93 (*p* < 0.001) at 6 months. Average operative time was similar in both groups, 76.41 and 79.15 min for group 1 and 2, respectively (*p* = 0.621).

**Conclusion:**

Our research with short-term results reports similar improvement in BMI and GERD symptoms in patients with LSG and HHR with or without LNF. A sound repair of hiatal crura combined with LSG leads to comparable outcomes to crural repair combined with LNF and LSG for weight loss and reflux resolution. Our short-term results do not support LNF in combination with LSG and HHR. Further research is essential to determine the long-term outcomes.

## 1 Introduction

Obesity and gastroesophageal reflux disease (GERD) are prevalent health concerns globally, often occurring concurrently and significantly impacting the quality of life of the affected patients (1). The prevalence of GERD in obesity ranges from 14.9% (2) to 24.2% (3). Laparoscopic sleeve gastrectomy (LSG) is the most commonly performed bariatric and metabolic surgery worldwide (4, 5). The usage of LSG has increased over the past years due to its comparable effectiveness in weight reduction and resolution of obesity-related type II diabetes mellitus, hypertension, and obstructive sleep apnea, as well as its short learning curve for surgical trainees (6). However, the development of *de novo* GERD after LSG has been increasingly reported (7). This relationship can be due to the changes in the anatomy of gastro-esophageal junction with dissection of angle of His and potential damage to the sling fibers of the lower esophageal sphincter, reduced gastric compliance after removal of gastric fundus, increased intragastric pressure as compared to intra-esophageal pressure, and, most importantly, the presence of hiatal hernia (8, 9). Nevertheless, the prevalence of 20%–60% of post-LSG GERD symptoms warrants attention, considering its negative impacts on the quality of life as well as the potential cellular changes associated with GERD and esophagitis carrying a risk of malignant transformation (10).

The prevalence of pre- and intra-operative hiatal hernias in obese populations remains as high as 18.9% (11). In addition, the contributions of hiatal hernias to GERD symptoms necessitate concurrent hiatal hernia repair (HHR) during bariatric procedures (12). There is a wealth of evidence that HHR during LSG can reduce postoperative GERD symptoms and improve patient comfort (13). Yet, the long-term efficacy and impact on overall surgical outcomes continue to be areas of active research. Furthermore, laparoscopic Nissen's fundoplication (LNF) is a well-established surgical therapy for GERD and has been demonstrated to provide effective and lasting relief from GERD symptoms (14). However, the adaptation of fundoplication techniques in conjunction with LSG and HHR is a subject of growing interest, which may potentially lead to a reduction in postoperative GERD symptoms (15).

Concurrently, HHR is frequently performed during LSG to address anatomical hernial defects contributing to GERD. Ther is a growing interest to investigate the outcomes of combining LSG, HHR, and LNF for the symptomatic relief of GERD and obesity (16). While adding LNF with HHR during LSG might be expected to increase operative time and potential complications, recent studies have varied in their findings, and have argued that with experienced surgical teams and improved techniques, these additions may not significantly impact the overall clinical outcome (12). The areas of concerns remain the careful creation of the fundal pouch for LNF which might be a source of weight regain in the long run. Currently, however, a great corpus of literature has shown a diversity of outcomes and underscores the need for careful patient selection and technique refinement (17).

Unfortunately, the outcome of adding LNF to LSG along with HHR is not fully understood. There remains an inadequacy of comparative studies and long-term follow-ups which can endorse a comprehensive understanding of the best surgical strategy for treating concurrent obesity and GERD either with LSG and HHR or incorporating LNF with LSG and HHR. This original clinical work is first of its kind in the Middle East and North Africa (MENA) region which is positioned at the intersection of surgical innovations and patient-centric care by comparing the reduction in body mass index (BMI) and GERD symptoms by LSG and HHR with or without LNF. Additionally, our work reports the operative time variations and the incidental finding of hiatal hernias during LSG.

## 2 Materials and methods

### 2.1 Study setting and design and

From January 2021 to February 2023, we conducted a retrospective clinical study to compare the effects of LSG and HHR, with or without LNF, on BMI and resolution of GERD symptoms. This study was conducted at Medcare Hospital, Dubai, United Arab Emirates, a high-volume bariatric and upper gastrointestinal surgery healthcare facility in Dubai, UAE. The primary aim of our study was to compare the operating time and BMI and GERD resolution in patients undergoing LSG with HHR, as opposed to LSG with HHR + LNF.

Data was collected retrospectively by accessing electronic medical records, and by conducting face-to-face and online interviews with the patients to retrieve their perceptions about the GERD Questionnaire (GERD-Q). A comprehensive data analysis was done to compare the two groups of patients: those who underwent LSG with HHR + LNF (group 1) and those who underwent LSG with HHR (group 2).

### 2.2 Patient population and inclusion and exclusion criteria

During the study period, all consecutive patients undergoing LSG with HHR with or without LNF were included in this research. To ensure a focused and relevant study population, we established specific inclusion and exclusion criteria for patients undergoing these surgical procedures. These criteria were designed to create a homogeneous group for analysis, thereby enhancing the validity and reliability of our findings so that they can be extrapolated to similar patient groups in clinical practice. Our study sample included patients of all ages and both genders, those with BMI meeting the criteria for bariatric surgery as per the Dubai Health Authority Standards for Bariatric Services, and a hiatal hernia (whether pre-operatively diagnosed or incidentally found during the operation) (18). As per the local bariatric guidelines, patients eligible for metabolic surgeries are those with BMI >35 kg/m^2^ or >30–34.9 kg/m^2^ along with poorly controlled type II diabetes mellitus or two obesity related diseases (hypertension, nonalcoholic fatty liver disease, polycystic ovary syndrome, dyslipidemia) (19). Patients included in the study had a minimum of 6 months follow-up after the surgical intervention. We excluded patients who lost follow-up within 6 months post-surgery, as well as those with incomplete medical records.

### 2.3 Evaluation tools

To assess the effectiveness of LSG and HHR, both with and without LNF, we employed a set of evaluation tools, which were used to assess the patients pre-operatively, and again at regular post-operative intervals.

#### 2.3.1 GERD questionnaire

In the outpatient clinic of the Medcare Hospital, all patients with morbid obesity and/or GERD symptoms were administered the GERD-Q (20), which served as a reliable diagnostic tool to ascertain the severity of the disease. The validated GERD-Q was selected as a primary tool for several reasons. The first reason was its consistency as, by using the same tool pre- and post-operative, we could directly compare symptom severity and assess the impact of the surgical intervention. Second, the specificity of GERD-Q was optimal as the questionnaire is tailored to capture the specific symptoms associated with GERD, making it an appropriate instrument for measuring changes related to our surgical interventions. Third, the instrument could be administered quickly and efficiently, making it feasible for routine clinical use and follow-up evaluations. We adopted the GERD-Q to determine the outcomes of surgical procedures in both groups. This tool has been widely used for the evaluation of therapies with promising results in terms of patient's perspectives about their conditions. The GERD-Q contains six questions focused on positive predictors (heartburn, regurgitation, difficulty sleeping due to symptoms, and the need for antacids) and negative predictors (epigastric pain and nausea). Each item was scored, contributing to a maximum score of 18, which represented the severity of GERD symptoms. The same GERD-Q was administered pre-operatively and at regular intervals (1, 3, and 6 months) post-operatively. This follow-up was conducted in the clinic or over the phone, ensuring a continuity and standardization in the evaluation of the patients' condition and the effectiveness of the surgical treatment.

#### 2.3.2 BMI measurement

BMI was recorded pre-operatively, and again during the regular post-operative follow-up visits at 1, 3 and 6 months after the surgery. BMI provides a standardized and objective evaluation of weight change measurement, mainly targeted to record an objective effectiveness of bariatric surgery (21).

##### 2.3.2.1 GERD and hiatus hernia

According to the hospital protocol, we performed pre-operative upper GI endoscopy for patients symptomatic of GERD, and per-operative endoscopy on asymptomatic patients to rule out hiatus hernia. We followed the surgical indications for GERD and hiatus hernia according to the Society of American Gastrointestinal and Endoscopic Surgeons guidelines (22). The implications of hiatal hernia were discussed with all patients scheduled for LSG, and the need of crural repair in case of hiatal hernia (whether pre-operatively diagnosed or found per-operatively) was explained to all patients. The option of LNF was offered to all patients whose hiatal hernia was found by a pre-operative endoscopy, and it was performed in those who consented for the procedure.

#### 2.3.3 Data collection and follow-up

The patient clinical data, including demographics, surgical history, BMI, and operation time were extracted from the electronic medical records (Mediware and TrakCare) of the hospital. By characterizing and understanding our patient population, we ensured that the findings of our study were grounded in a robust and representative sample, thereby enhancing the generalizability and applicability of our conclusions.

##### 2.3.3.1 Surgical technique

**Figure 1:**
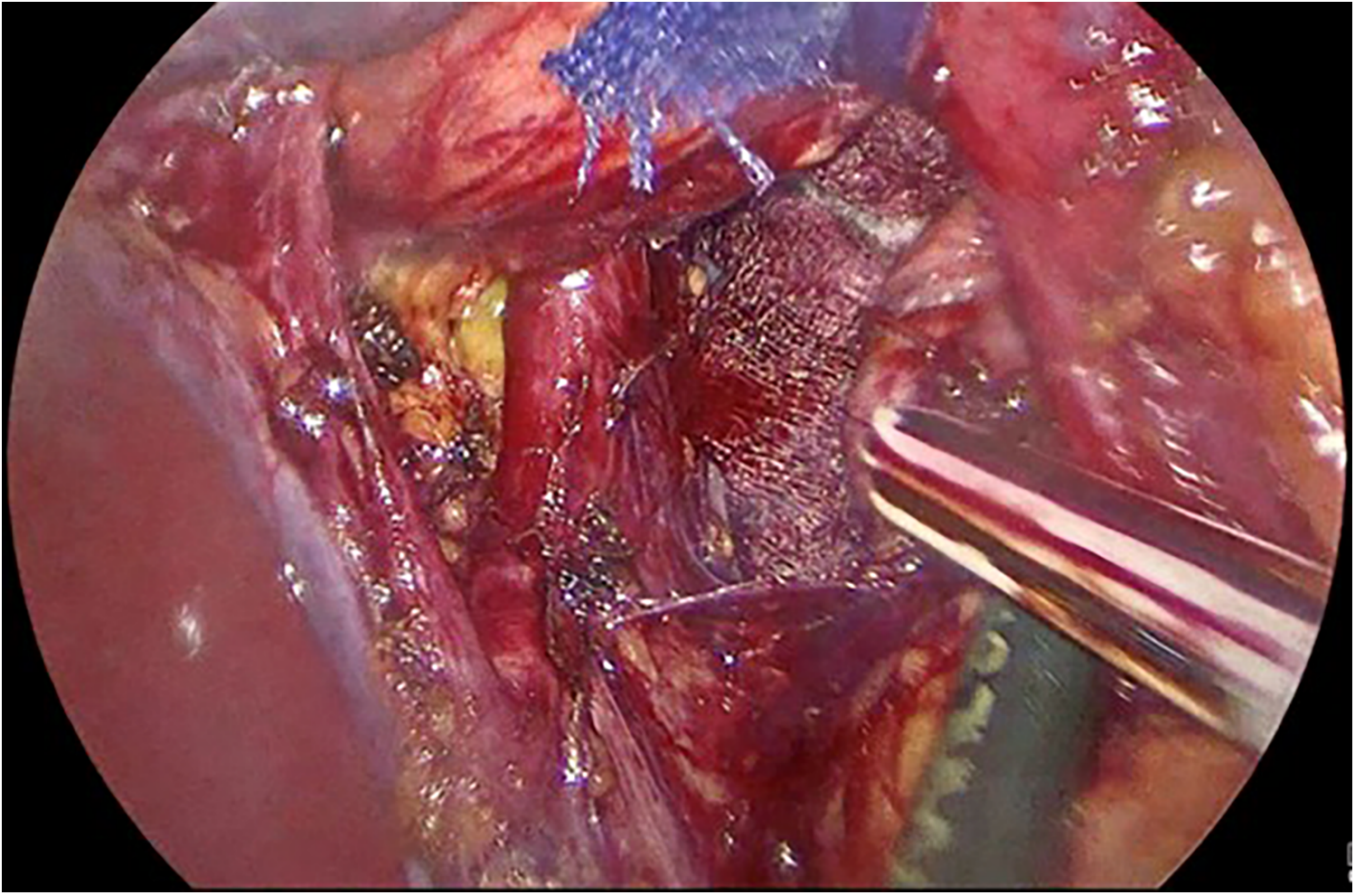
Hiatal hernia defect clearly visible after surgical dissection.

**Figure 2:**
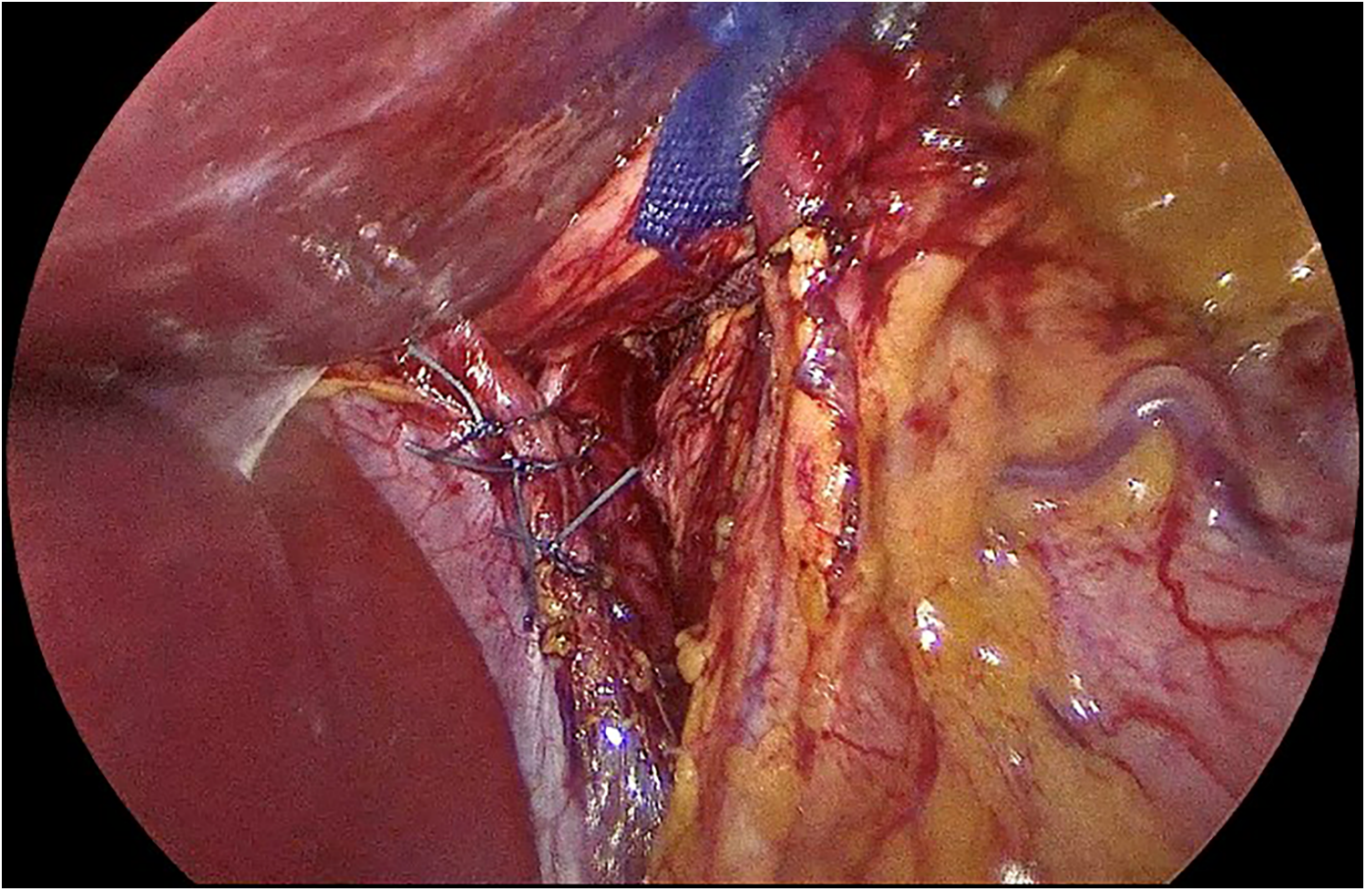
Repair of hernial defect using Ethibond sutures with a figure-of-eight technique.

Surgical technique performed was uniform for all the patients in the study sample. A laparoscopic approach was taken in all cases. With the patient placed in modified lithotomy position, access into the abdominal cavity was gained using an optic 5 mm trocar inserted at the left palmer point. Two operating ports, along with two retraction ports and a camera port were used in all patients. The greater omentum was dissected away from the stomach starting at 4 cm away from the pylorus, and up to the level of the cardia. Clear visualization of the left crus was ensured before moving on to opening the pars flaccida of the lesser omentum to gain access to the right diaphragmatic crus. Meticulous dissection was then carried out to reduce the stomach back into the abdominal cavity, achieving an abdominal esophageal length of at least 3 cm in all patients. A boogie was always used for the calibration of sleeve gastrectomy, which was introduced after fundoplication. Hiatal hernia defect was then repaired using Ethibond sutures (Figures 1, 2).

**Figure 3:**
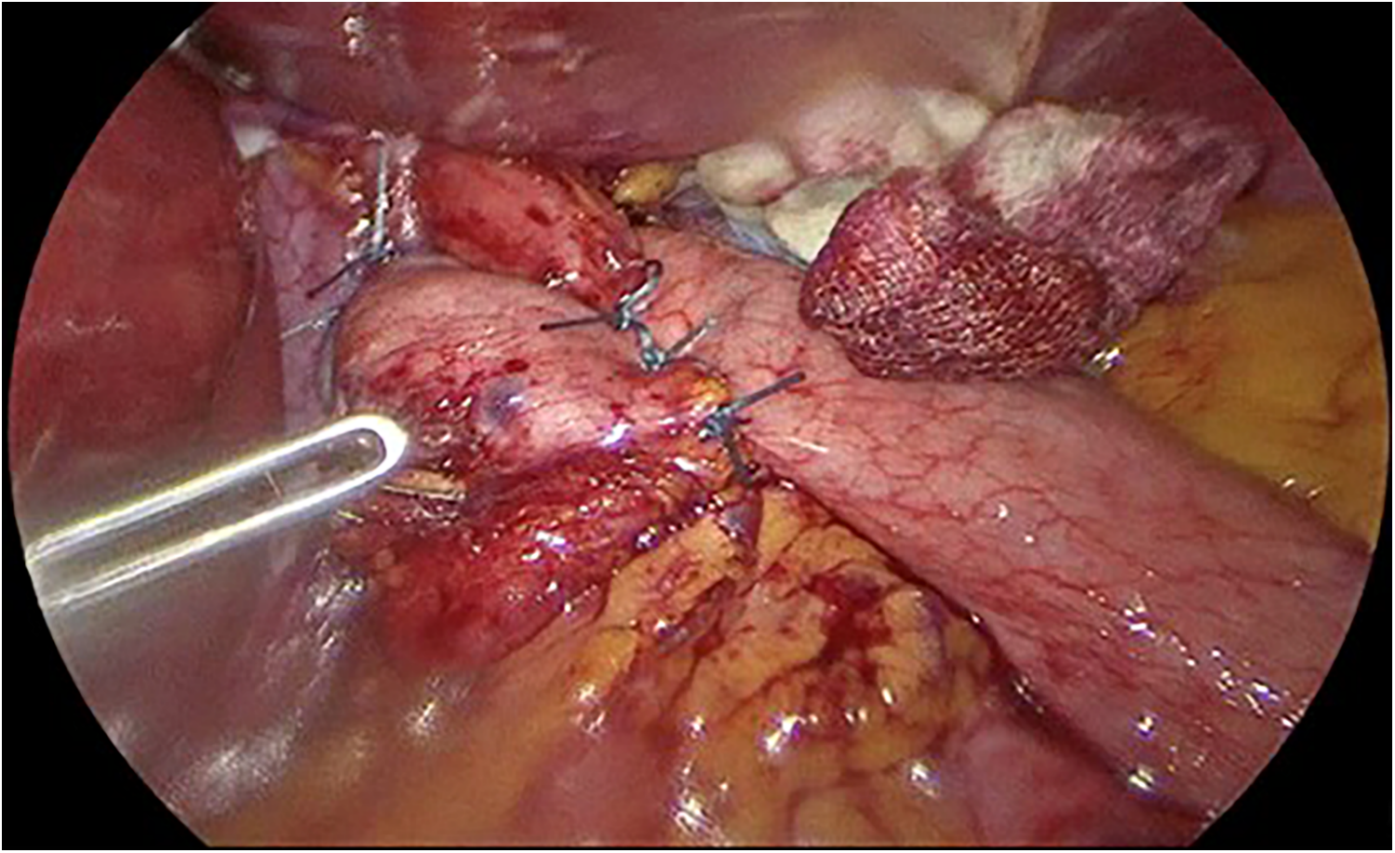
Laparoscopic Nissen fundoplication with a fundal wrap around lower esophagus.

Later LNF was performed after the crural repair on patients in group 1 (Figure 3).

**Figure 4:**
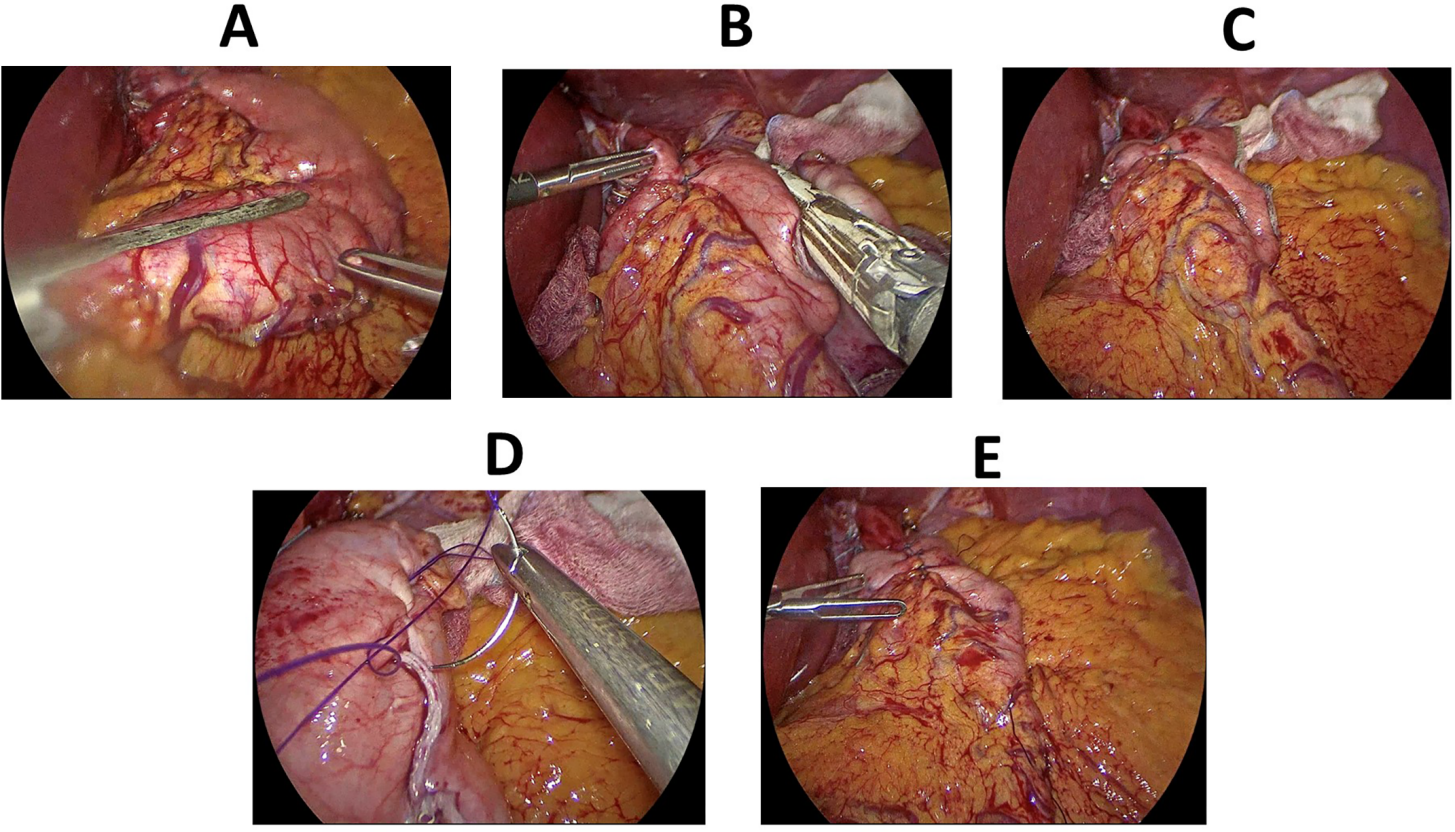
Key steps of laparoscopic sleeve gastrectomy. **(A)** Sleeving of the stomach, starting approx. 4 cm away from the pylorus. **(B)** Sleeving of the stomach continued upwards sparing the fundoplication. **(C)** A surgical view of gastric sleeve with the hiatal hernia repair and fundoplication. **(D)** Oversewing the staple line after the creation of the gastric sleeve. **(E)** A final surgical view after oversewing the staple line and fixing the sleeved stomach to the omentum.

Stomach was then sleeved using EndoGIA linear staplers, starting at 4 cm away from the pylorus and up to the level of cardia, preserving the fundoplication in group 1 patients (Figures 4A–E).

Finally, the staple line was buried using continuous PDS or Stratafix sutures and an on-table endoscopy with leak test was performed.

The same surgical team was involved in the surgical management of the patients before and during the study period which enhances the consistency and accuracy of clinical outcomes of this work.

#### 2.4 Statistical analysis

We conducted a comprehensive statistical analysis using the open-source software Jamovi 2.3.28.0. Descriptive statistics were computed to provide an overview of the data, including means, standard deviations, and frequencies for relevant variables. Comparative analyses were conducted using independent samples *t*-tests to compare pre-operative and post-operative BMI, duration of surgery, and GERD-Q scores between patients who underwent LSG, HHR with LNF (group 1) and those who underwent the procedure without LNF (group 2).

For the evaluation of the impact of surgical procedures, we conducted both parametric and non-parametric statistical analyses. An independent sample *t*-test was utilized to compare the means of surgery, accommodating for normally distributed data. However, due to the significant variance in group sizes as indicated by Levene's test, we further employed the Mann–Whitney *U* test. This non-parametric test allowed us to compare the medians of two groups without the assumption of normal distribution or equal variances, providing a robust analysis of the differences in surgery duration. Both tests were conducted at a 95% confidence level, and the results were reported in terms of test statistics, degrees of freedom, and p-values. Additional analyses included paired *t*-tests to compare GERD-Q scores before and after surgery within each group and correlation analyses to explore the relationships between the duration of surgery and post-operative outcomes.

Throughout the analysis, appropriate visualizations were generated to aid in the interpretation and presentation of the data. For the visualization of the computed correlations, the seaborn 0.11.2 library was utilized. A heatmap was generated using seaborn's, providing an intuitive color-coded representation of the correlation matrix (23). The colormap “coolwarm” was employed to signify the spectrum of correlation values, with warmer colors indicating stronger positive correlations and cooler colors representing negative correlations. For enhanced interpretability, the correlation values were formatted to two decimal places and annotated directly onto the heatmap. Correlation analysis was conducted in a Python environment. The matplotlib 3.4.3 library supported additional customization of the visualizations, enhancing the aesthetic and functional aspects for clearer interpretation.

#### 2.5 Ethical approval

This study was approved by the Research Ethics Committee at Medcare Hospital, Dubai, UAE (Ref MCH/ETHCS/003). Informed consent was obtained from all patients involved in this research.

## 3 Results

During the study period, our facility performed 978 bariatric surgeries including LSG, laparoscopic Roux-en-Y gastric bypass, laparoscopic adjustable gastric banding, and laparoscopic one-anastomosis gastric bypass. Of 431 cases of LSG, seven patients were lost in follow up and 73 patients fulfilled our study criteria. These included 32 patients in group 1 and 41 in group 2. There were 57 women and 16 men with a mean age of 41 years. Furthermore, 26 of the hiatal hernias were discovered per-operatively which were surgically repaired during LSG, all without subsequent fundoplication.

### 3.1 Descriptive analysis

In examining the pre-operative and post-operative parameters of patients undergoing surgical procedures, distinct patterns emerged between the two groups as shown in Table 1.

**Table 1:** Descriptive statistics for pre- and postoperative BMI, GERD-Q, and duration of surgery for patients in group 1 and 2 (*N* = 73).

Group 1: LSG + HHR + LNF^a^							
	Pre-op BMI	Pre-op GERD-Q	Surgery duration	Post-op BMI (1 mon)	Post-op BMI (3 mon)	Post-op BMI (6 mon)	Post-op GERD-Q
*N*	32	32	32	32	24	30	32
Missing	0	0	0	0	8	2	0
Mean	38.0	12.3	76.4	35.4	32.8	32.2	6.47
Median	37.0	12.0	72.5	35.0	32.0	31.5	6.00
Standard deviation	5.40	2.18	21.5	4.81	4.98	4.75	1.54
Minimum	28	7	45	27	25	23	3
Maximum	48	15	160	45	42	43	12
Group 2: LSG + HH^a^							
*N*	41	41	41	41	35	39	41
Missing	0	0	0	0	6	2	0
Mean	39.6	11.5	79.1	36.8	34.2	31.7	6.93
Median	38	12	70	36	34	31	6
Standard deviation	5.04	2.78	24.7	5.13	5.30	4.94	1.75
Minimum	31	6	40	26	26	25	3
Maximum	50	15	150	47	45	43	12

^a^ LSG + HHR + LNF laparoscopic sleeve gastrectomy + hiatal hernia repair + laparoscopic Nissen fundoplication.

In group 2 with 41 patients, the average pre-operative BMI of 39.63 was recorded with a subsequent BMI decrease at 1-month (36.78), 3-months (34.20), and 6-months (31.67) post operatively. The pre-operative GERD-Q scores averaged at 11.54, with a notable reduction to 6.93 post-operation. The duration of surgery for this group varied, with an average time of 79.15 min.

Group 1, with a sample of 32, demonstrated a slightly lower average pre-operative BMI of 38.03, which declined at 1-month (35.44), 3-months (32.79), and 6-months (32.17) during follow-ups. The pre-operative GERD-Q scores were marginally higher in this cohort, averaging at 12.25, but showed a significant decrease to 6.47 post-procedure. The average duration of surgery was slightly shorter at 76.41 min.

### 3.2 Comparative analysis

The comparative analysis of results using the independent samples *t*-test showed no significant differences between the groups at any time point (Table 2). At 1 month, the t-statistic was 1.14 (*p* = 0.258); at 3 months, 1.03 (*p* = 0.308); and at 6 months, −0.42 (*p* = 0.673). The findings of the Levene's Test for equality of variances supported these findings, indicating similar variances between groups at each interval (*p* = 0.990 at 1 month, *p* = 0.657 at 3 months, and *p* = 0.544 at 6 months). During the evaluation of GERD-Q scores, both before and after surgery, no significant differences were found. The statistical analysis showcased a t-statistic of −1.19 (*p* = 0.238) pre-operation and 1.17 (*p* = 0.247) post-operation, indicating a lack of statistical significance. Levene's test corroborated the consistency of variances between the groups, with *p*-values of 0.078 and 0.382 for pre- and post-operation, respectively.

**Table 2:** A comparison of post-operative BMI reductions at various intervals and GERD-Q scores, pre- and post-operation, and duration of surgical procedures between two groups using *t*-test (*N* = 73).

Post-operative BMI reductions				
		Statistic	df	*p*
Post-op BMI (1 mon)	Student's *t*	−1.141	71.0	0.258
Post-op BMI (3 mon)	Student's *t*	−1.028	57.0	0.308
Post-op BMI (6 mon)	Student's *t*	0.424	67.0	0.673
Homogeneity of variances test (Levene's)				
	F	df	df2	*p*
Post-op BMI (1 mon)	1.53e-4	1	71	0.990
Post-op BMI (3 mon)	0.200	1	57	0.657
Post-op BMI (6 mon)	0.372	1	67	0.544
GERD-Q scores pre-operation and post-operation				
		Statistic	df	*p*
Pre-op GERD-Q	Student's *t*	1.19	71.0	0.238
Post-op GERD-Q	Student's *t*	−1.17	71.0	0.247
Homogeneity of variances test (Levene's)				
	F	df	df2	*p*
Pre-op GERD-Q	3.195	1	71	0.078
Post-op GERD-Q	0.775	1	71	0.382
Duration of surgery				
		Statistic	df	*p*
Duration of surgery	Student's *t*	−0.497	71.0	0.621
Homogeneity of variances test (Levene's)				
	F	df	df2	*p*
Duration of surgery	1.61	1	71	0.208

The comparison of the duration of surgical procedures between groups also showed no significant disparity. The independent samples *t*-test offered a t-statistic of 0.50 (*p* = 0.621), suggesting a lack of meaningful difference. This implication was further supported by Levene's test, which presented a statistic of 1.61 and a *p*-value of 0.208, indicating uniformity in the variance across the groups.

The results of the paired *t*-tests for the intra-group comparisons objectively assessed the mean differences between pre- and post-operative values of GERD-Q scores and BMI at various intervals are displayed in Table 3. For group 2, significant reductions were observed in both GERD-Q scores (*t* = 8.38, *p* < 0.001) and BMI across all post-operative checkpoints; 1 month (*t* = 9.53, *p* < 0.001), 3 months (*t* = 18.76, *p* < 0.001), and 6 months (*t =* 17.06, *p* < 0.001). A similar trend was identified in group 1, with notable decreases in GERD-Q scores (*t* = 14.33, *p* < 0.001) and BMI at 1 month (*t* = 10.57, *p* < 0.001), 3 months (*t* = 11.07, *p* < 0.001), and 6 months (*t* = 10.00, *p* < 0.001). These results indicate substantial improvements in both GERD symptoms and BMI within the first 6 months after surgery, irrespective of the fundoplication procedure.

**Table 3:** Comparative analysis of pre- and post-operative GERD-Q scores and BMI changes at different time points using paired *t*-tests in both groups (*N* = 73).

	Group 1: LSG + HHR + LNF^a^				
			statistic	df	*p*
Pre-op GERD-Q	Post-op GERD-Q	Student's *t*	14.33	31.0	**<.001**
Pre-op BMI	Post-op BMI (1 m)	Student's *t*	10.57	31.0	**<.001**
	Post-op BMI (3 m)	Student's *t*	11.07	23.0	**<.001**
	Post-op BMI (6 m)	Student's *t*	10.00	29.0	**<.001**
Group 2: LSG + HHR^a^					
Pre-op GERD-Q	Post-op GERD-Q	Student's *t*	8.38	40.0	**<.001**
Pre-op BMI	Post-op BMI (1 m)	Student's *t*	9.53	40.0	**<.001**
	Post-op BMI (3 m)	Student's *t*	18.76	34.0	**<.001**
	Post-op BMI (6 m)	Student's *t*	17.06	38.0	**<.001**

Bold indicates significant values (*p* < 0.001).

^a^ LSG + HHR + LNF laparoscopic sleeve gastrectomy + hiatal hernia repair + laparoscopic gastric fundoplication.

### 3.3 Correlation analysis

**Figure 5:**
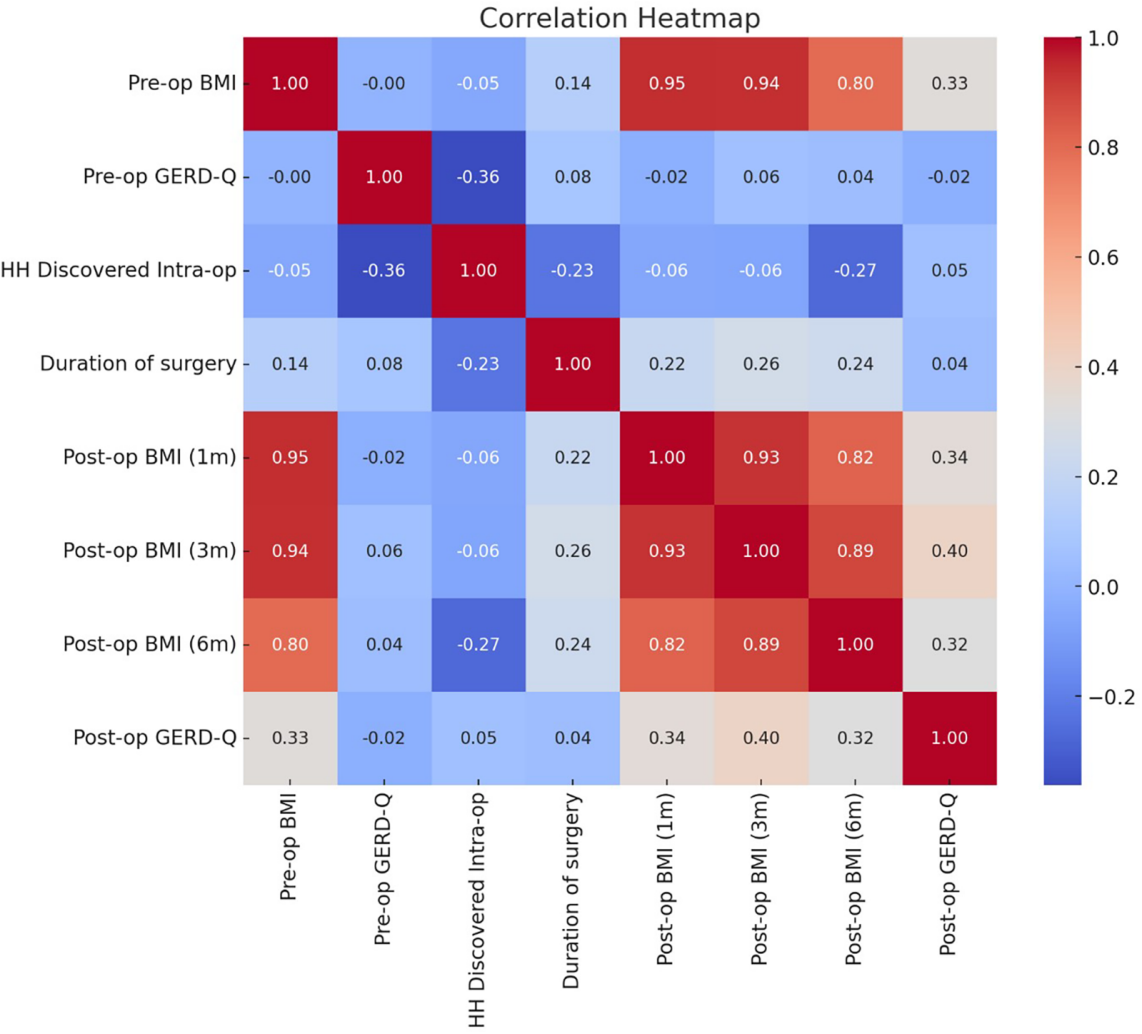
A heatmap analysis for the correlation dynamics in postoperative outcomes.

Upon computation of the pairwise correlations, the analysis yielded a comprehensive matrix encompassing all continuous and binary variables. Each coefficient within this matrix represented the correlation between two distinct variables, providing a quantitative measure of their relationship's strength and direction. The correlation matrix's complexities were distilled into a color-coded heatmap (Figure 5), offering a visually intuitive representation of the dataset's statistical relationships.

This graphical approach facilitated a clear distinction between variables, employing a “coolwarm” color spectrum to indicate the range of correlation values. In the heatmap, each variable pair was represented by a cell, the color of which signified the correlation value, with the actual numeric coefficient annotated within. The color intensity increased with the absolute value of the correlation, with the “cool” end of the spectrum (blues) representing negative correlations, transitioning through neutral (whites) for negligible or no correlation, and intensifying into the “warm” end (reds) for positive correlations.

The heatmap displayed several notable features; the range of correlation values spanned from −1 to 1, indicating the presence of both negative and positive correlations in the dataset. The findings from the heatmap included the post-op BMI measurements which showed strongly positive correlations among post-op BMI (1 m), post-op BMI (3 m), and post-op BMI (6 m). For the intra-operative discovery of hiatus hernia, there were variable correlations with other outcomes, suggesting the impact of intraoperative finding on post-operative outcomes. Lastly, the analysis showed a moderately positive correlation with post-op BMI, suggesting a linkage between high BMI and worse GERD symptoms post-surgery.

### 3.4 Complications

The study reported three complications, with a complication rate of 4.1%. A woman from group 1 presented on 6th post-operative day with fever, abdominal pain, and diarrhea. A CT-scan of the abdomen showed intraperitoneal collections in the perigastric and perisplenic, and pelvic regions. A diagnostic laparoscopy was done, and an intra-operative endoscopy showed a positive leak test. A clear leak point could not be detected, and an abdominal drain was placed after a peritoneal lavage. Gastrografin swallow under fluoroscopy was done on 4th post-operative day, which did not show leak of contrast. Patient tolerated clear liquid diet and was discharged in a stable condition. Second patient, from group 1, returned to the ER on 2nd post-operative day, with complaints of continuous vomiting and dehydration. A gastrografin swallow under fluoroscopy showed an obstruction at the level of fundoplication, with contrast stopping at the lower end of esophagus. Endoscopy showed a twist in the fundoplication. Laparoscopic surgery was performed to undo the fundoplication. The patient tolerated clear liquid diet and was discharged in stable condition. Lastly, another patient from group 1, on her 6-month follow-up, reported resolution of GERD symptoms, but she only lost 6 Kg. All her labs and hormonal values were normal. A fluoroscopic imaging was done for the evaluation of the stomach volume. Then, a subsequent laparoscopic surgery showed a dilated fundus, which necessitated refashioning of the sleeve gastrectomy, without undoing fundoplication. She reported 7 kg weight loss on her 1-month follow-up after re-operation.

## 4 Discussion

This study demonstrates short-term results of the clinical outcomes of the postoperative BMI and GERD symptoms, after concurrent integration of LNF with LSG and HHR. Both groups showed significant reductions in BMI and GERD-Q scores post-surgery (*p* < 0.001), while there was insignificant difference in the average operative time (*p* = 0.621). This unique work entails that the concurrent addition of LNF to LSG with HHR has comparable clinical improvements in BMI and GERD symptoms to those with only LSG and HHR. However, our short-term results need validation by long-term follow up to optimize patient selection and sustainable management strategies for morbid obesity and GERD symptoms.

By examining various clinical parameters including BMI changes, GERD-Q scores, and operative times, our work has provided a comprehensive evaluation of LSG, HHR, and LNF for patients with obesity and hiatal hernia. In a systematic review and meta-analysis, Lidia et al., have elucidated that both LSG with concomitant HHR and LNF remained effective for reflux resolution and weight loss outcomes, with superiority of LSG and fundoplication for GERD improvement, despite a higher rate (12). However, we found that adding LNF to LSG with HHR has similar short-term outcomes, in terms of GERD-Q scores and BMI improvements, to those after LSG and HHR alone, without any significant increase in operative time.

A study on the short-term results of LSG with fundoplication for GERD symptoms and obesity has shown optimal results in both reflux resolution and weight loss (24). However, during the long-term follow-up, six patients underwent revision surgery, five for weight regain and one for reflux symptoms. In our short-term follow up, no weight regain has been reported yet, but weight regain is an established complication of LSG with LNF due to the dilatation of the fundal pouch and its associated hormonal change (25). From a different perspective, Jing et al., have argued that LSG alongside fundoplication achieves better GERD remission but leads to poor weight loss and increased postoperative complications compared with LSG alone (14). In contrast, in our short-term results, BMI reduction and GERD improvement remained significant in both groups. This may be attributed to a special attention to the surgical technique especially the sleeving of the stomach with the preservation of a floppy fundal wrap.

In our study, the analysis of the clinical outcomes of patients in both groups unveils critical insights into the efficacy and impact of the surgical procedures. Notably, both groups exhibited a consistent reduction in BMI post-surgery, indicative of the short-term effectiveness of the surgical interventions. However, the slightly lower initial BMI and the marginally higher pre-op GERD-Q scores in group 1 highlight a critical interplay of the effectiveness of surgical procedures in mitigating GERD symptoms, irrespective of the initial symptom severity. Interestingly, the average duration of surgery did not differ significantly between groups, although it was reported in literature that fundoplication procedure might extend operative time (24). This could imply a level of surgical expertise in mitigating potential time-related risks. The role of surgical training inside and outside of the operating room plays substantial roles in upskilling the surgical techniques (26, 27). The duration of surgery, an aspect critical to assessing procedural efficiency and resource allocation, did not differ significantly between both groups.

The post-operative assessments in our study indicate a consistent trend in BMI reduction in both groups. This similarity suggests that the addition of fundoplication does not markedly influence the trajectory of weight loss, at least in the early post-operative phases. As rightly pointed out by Potrizio et al., the weight loss trend post bariatric surgery does not reach clinical goal within 2 years, and a sustainable and reliable weight loss achievement should be reported after a medium and long-term follow-up (28). This finding reaffirms that the timepoint of post-surgery follow-up seems to be a reliable predictor that may influence the loss of therapeutic success in case of weight regain.

As quantified by the GERD-Q scores, our study findings depicted an insignificant difference between groups which indicate that fundoplication does not significantly alter GERD-related symptoms. The homogeneity in variances underscores similarity in groups, indicating that both sets of patients, reported similar degrees of symptomatology. The outcomes from the intra-group comparisons elucidated compelling trajectories in patient recovery and symptomatology following bariatric surgery, contingent upon the inclusion of fundoplication. Both cohorts manifested pronounced improvements with substantial reductions in GERD-Q scores and BMI at successive post-operative intervals. In group 1, the addition of fundoplication seemed to bolster optimal outcomes, aligning with existing literature that posits fundoplication as a beneficial adjunct to standard bariatric procedures, fortifying defenses against reflux symptoms (29). A marked decrease in GERD-Q scores in our study signals the potential of combined procedures in offering symptomatic relief. However, group 2 patients, despite the absence of LNF, did not lag in post-operative improvements, suggesting that standard LSG along with HHR bear considerable merit in addressing both obesity and GERD.

Although GERD-scores are universally adopted to determine the severity of reflux symptoms, literature has shown that 24-h pH study, high-resolution manometry, and upper GI endoscopy are essential diagnostic tools for a thorough evaluation of GERD (20). We followed the standard hospital policy by using the GERD-Q for the evaluation of reflux symptoms and so far, the patients' symptoms closely corroborate with their GERD scores. The persistence of GERD symptoms postoperatively for some patients, as evidenced by the post-op GERD-Q scores, suggests that while surgical interventions may alleviate certain manifestations of GERD, it is not a panacea, particularly for more severe or persistent cases (30). The existing body of literature has shown that preoperative BMI serves as reliable predictor of shorter-term weight loss outcomes following bariatric procedures, however, it should not be considered in isolation when the quality of bariatric surgery is being evaluated (31). The complexity of bariatric surgery procedures along with obesity comorbidities underscores the necessity for a holistic approach to patient management, by integrating interprofessional collaboration strategies (32), and by adopting the recommended surgical guidelines to optimize post-operative outcomes (33). In our study, the heatmap of statistical correlations identified several intriguing interrelationships between patient metrics, particularly highlighting the interconnected nature of post-operative BMI at various post operative intervals. Notably, the strong positive correlations between post-op BMI, at 1-month, 3-month, and 6-month highlight a consistent trajectory of weight loss post-surgery.

### 4.1 Study limitations

This study reports short-term clinical outcomes on a small group of non-randomized patients. More longitudinal studies on a large dataset of patients are essential to validate this study results. Long-term follow-up is needed for optimization of patient selection and improvement of management strategies. Additionally, while the statistical significance confers clinical relevance, it is imperative to consider that these findings rely on self-reported GERD-Q scores which might impact the elements of subjective bias, necessitating corroboration with objective diagnostic assessments.

## 5 Conclusion

Our research reports similar improvement in BMI and GERD symptoms when comparing patients with LSG and HHR with or without LNF. However, a sound crural repair is essential in patients with LSG and HHR alone which may obviate the need of LNF. LSG is a feasible bariatric option in those patients with hiatus hernia, as long as the hernia is repaired. However, we consider this research to be a first step in investigating the effects of LNF, on BMI and GERD, when combined with LSG and HHR. We believe that following long-term outcomes, especially those regarding weight regain, to be an area of great interest and potential investigative value, always with the aim of optimizing patient selection and improving management strategies when caring for patients with morbid obesity and GERD symptoms.

## Data Availability

The raw data supporting the conclusions of this article will be made available by the authors, without undue reservation.
